# Hybrid repair of complicated acute aortic arch intramural haematoma with the Castor single-branch stent graft

**DOI:** 10.1093/icvts/ivae163

**Published:** 2024-10-15

**Authors:** Antonio Rizza, Cataldo Palmieri, Silvia Di Sibio, Michele Murzi

**Affiliations:** Cardiology Unit, Heart Hospital, Fondazione Toscana “G. Monasterio”, Massa, Italy; Cardiology Unit, Heart Hospital, Fondazione Toscana “G. Monasterio”, Massa, Italy; Institute of Life Sciences, Scuola Superiore Sant’Anna, Pisa, Italy; Adult Cardiac Surgery Unit, Heart Hospital, Fondazione Toscana “G. Monasterio”, Massa, Italy

**Keywords:** Acute aortic syndrome, Endovascular, Aortic arch

## Abstract

We report the off-label application of the Castor single-branch stent graft for a complicated acute intramural haematoma involving the aortic arch. The endograft was deployed in zone 1 with the single branch in the left common carotid artery through a surgical left carotid and percutaneous right femoral artery access. The procedure was completed with the construction of a left carotid-subclavian bypass followed by plug embolization of the left subclavian artery.

## CASE PRESENTATION

**Figure 1:**
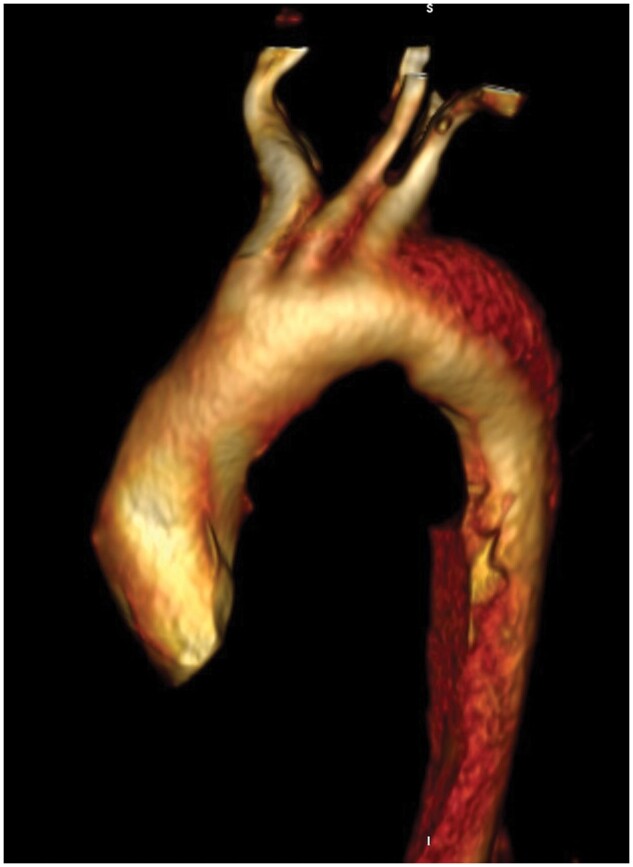
3-Dimensional computed tomography angiographic reconstruction shows an acute intramural aortic haematoma extending from the distal aortic arch to the abdominal aorta.

Α 58-year-old woman presented to our facility with a diagnosis of aortic intramural haematoma (IMH) extending from the distal aortic arch to the renal arteries (Fig. 1). Her medical history was remarkable for hypertension and hyperlipidaemia. Initially, medical treatment with analgesia and blood pressure and heart rate monitoring was adopted. However, despite 72 h of pharmacological management, the patient continued to report uncontrolled pain. After an aortic team discussion, an emergency hybrid procedure, consisting of revascularization of the supra-aortic vessels followed by endograft delivery, was chosen. To reduce the invasiveness of the procedure and considering that zone 1 was completely free from disease, we decided to use a preloaded branched endograft (Castor, Shanghai Microport Endovascular MedTech, Shanghai, China) to be deployed in zone 1 with the branch in the left common carotid artery (LCCA) and a concomitant left carotid-subclavian artery bypass.

## PROCEDURE

**Figure 2:**
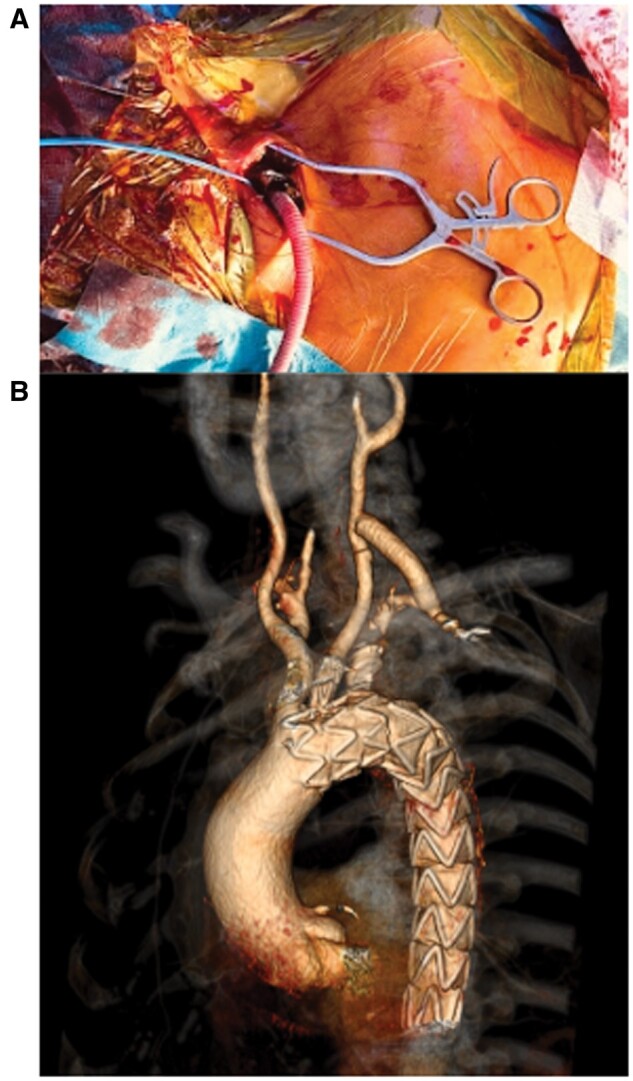
(**A**) Vascular access was created through an 8-mm Dacron graft directly anastomosed to the LCA. The Dacron graft was used at the end of the procedure to create a left carotid-subclavian bypass. (**B**) Computed tomography angiography scan taken at 3 months.

The procedure was performed with the patient under general anaesthesia; a 6 Fr pigtail was advanced through a right radial arterial access into an ascending aorta for aortic angiography; then 10 Fr right femoral arterial access was obtained with the pre-implantation of 2 percutaneous closure systems, subsequently upgraded to 18 Fr. The LCCA and the left subclavian artery (LSA) were exposed consecutively through a neck incision. An 8-mm Dacron graft was anastomosed to the LCCA and used as the vascular access (Fig. 2A). A guidewire was advanced through the LCCA access into the femoral access, forming a carotid–femoral loop; a dedicated catheter was advanced on the guidewire; then, an extra-stiff guidewire (0.035″) was advanced through the femoral access to the ascending aorta; the branch guidewire was positioned in the catheter introduced from the carotid access; and a Castor 34 mm × 30 mm × 200 mm stent graft with a 12 mm × 25 mm branch for LCCA was positioned and deployed (Fig. 2B and C, Supplementary Material, Video S1). Immediate aortography confirmed the patency of the side branch and the absence of endoleaks (Supplementary Material, Videos S2 and S3). The 8-mm Dacron graft was anastomosed to the LSA for the carotid subclavian bypass. Finally, a vascular plug (Amplatzer; AGA Medical Corp, Golden Valley, MN, USA) was advanced through the brachial artery and deployed at the origin of the LSA. The patient had an uneventful recovery and was discharged on postoperative day 10. At 3 months, the computed tomography angiography scan showed the absence of endoleak and the correct positioning and expansion of the side branch into the LCCA (Fig. 2B).

## DISCUSSION

The Castor single-branch stent graft is an endoprosthesis specifically designed to be deployed in zone 2 to preserve the LSA using a branch section [1]. It is made of a main body and a side that originates 5 to 30 mm from the proximal edge of the prosthesis. Many studies from China have reported good short- and long-term outcomes [2]. However, this device has rarely been used in Europe [3]. In our centre, we started to use the Castor graft in 2021; since then, we have treated an increasing number of patients with encouraging results [1, 4]. In the present case, we decided to use the Castor with an off-label indication, releasing the graft in zone 1 to cover all of the diseased arch while preserving the LCCA. This step allows us to avoid a first surgical step, such as arch debranching or a carotid-to-carotid bypass while obtaining an adequate proximal landing zone. The release of the side branch into the LCCA appeared to be technically simpler and faster than the usual release inside the LSA, because the carotid artery tends to take off in a direction that is more perpendicular to the aortic arch. Moreover, the use of an 8-mm Dacron graft directly anastomosed to the LCCA as the vascular access allows us to avoid retrograde puncture of the left carotid artery while obtaining easier and more navigable access for the carotid-femoral loop required for the branch release. Whether the LSA should be revascularized during a TEVAR procedure is still a matter of debate. We strongly believe that LSA revascularization has some advantages regarding neurological complications and left arm ischaemia. Although some authors have reported good results with in situ or in vitro fenestration of the LSA [5], we decided to perform a carotid-to-subclavian bypass for LSA revascularization, using the Dacron graft previously anastomosed to the LCCA. Indeed, both in situ and in vitro fenestration of the LSA are the most challenging of the 3 aortic arch branches due to the LSA anatomical form and take-off while the carotid-to-subclavian bypass is a simple surgical procedure that can be performed with a low risk of complications. In conclusion, releasing the Castor graft in zone 1 with the side branch into the LCCA is technically feasible and might represent a valid alternative in selected patients.

## Supplementary Material

ivae163_Supplementary_Data

## Data Availability

All relevant data are within the manuscript and its Supporting Information files.

## ABBREVIATIONS

LCCA: Left common carotid artery
LSA: Left subclavian artery
